# Accuracy of genotype imputation in Labrador Retrievers

**DOI:** 10.1111/age.12677

**Published:** 2018-07-05

**Authors:** J. Friedrich, R. Antolín, S. M. Edwards, E. Sánchez‐Molano, M. J. Haskell, J. M. Hickey, P. Wiener

**Affiliations:** ^1^ Division of Genetics and Genomics The Roslin Institute and Royal (Dick) School of Veterinary Studies University of Edinburgh Midlothian EH25 9RG UK; ^2^ Animal and Veterinary Sciences Group Scotland's Rural College Edinburgh EH9 3JG UK

**Keywords:** genome‐wide association studies, genomic prediction, imputation accuracy, low‐density array design, pedigree information, reference set

## Abstract

The dog is a valuable model species for the genetic analysis of complex traits, and the use of genotype imputation in dogs will be an important tool for future studies. It is of particular interest to analyse the effect of factors like single nucleotide polymorphism (SNP) density of genotyping arrays and relatedness between dogs on imputation accuracy due to the acknowledged genetic and pedigree structure of dog breeds. In this study, we simulated different genotyping strategies based on data from 1179 Labrador Retriever dogs. The study involved 5826 SNPs on chromosome 1 representing the high density (HighD) array; the low‐density (LowD) array was simulated by masking different proportions of SNPs on the HighD array. The correlations between true and imputed genotypes for a realistic masking level of 87.5% ranged from 0.92 to 0.97, depending on the scenario used. A correlation of 0.92 was found for a likely scenario (10% of dogs genotyped using HighD, 87.5% of HighD SNPs masked in the LowD array), which indicates that genotype imputation in Labrador Retrievers can be a valuable tool to reduce experimental costs while increasing sample size. Furthermore, we show that genotype imputation can be performed successfully even without pedigree information and with low relatedness between dogs in the reference and validation sets. Based on these results, the impact of genotype imputation was evaluated in a genome‐wide association analysis and genomic prediction in Labrador Retrievers.

## Introduction

The genetic, physiological and behavioural features of domestic dogs make them a valuable animal model for the genetic analysis of complex traits in genome‐wide association studies (GWAS) (van Steenbeek *et al*. 2016) that are also of interest in humans (Boyko 2011; Machiela & Chanock 2014; Hayward *et al*. 2016). Furthermore, the continuing popularity of pet dogs and concern for their welfare demand advanced breeding strategies like the use of genomic selection to improve animal health, maintain breed standards and control inbreeding. Establishing genomic selection in pedigreed dogs would generally require genome‐wide genotyping of large numbers of pet dogs. However, costs of genotyping can be a limiting factor as has been shown in studies of livestock species (Anderson *et al*. 2008; Huang *et al*. 2012; Gualdrón Duarte *et al*. 2013). Huang *et al*. (2012), for example, estimated costs for genotyping using a combination of low‐density (LowD) and high‐density (HighD) SNP genotyping arrays as ranging from $20.58 to $34.84 per individual compared to $120 per individual when genotyping all individuals at HighD. A LowD array is not currently available in dogs, but the financial benefit of using LowD arrays instead of HighD arrays is likely to be similar to that for other species.

Using LowD SNP genotyping arrays however leads to a loss of genotype information in comparison to a HighD array. To increase the cost effectiveness of genotyping, genotype imputation can be used to infer higher density genotypes, as has been shown in livestock species (Gualdrón Duarte *et al*. 2013; Carvalheiro *et al*. 2014; Boison *et al*. 2015; Ventura *et al*. 2016). Several approaches are available for imputing genotypes; they can be categorized primarily as methods using linkage disequilibrium (LD) alone [e.g. impute2 (Howie *et al*. 2009), beagle (Browning & Browning 2009) and mach (Li *et al*. 2010)] and methods using both LD and pedigree information [e.g. findhap (VanRaden *et al*. 2011), alphaimpute (Antolín *et al*. 2017) or fimpute (Sargolzaei *et al*. 2014)]. Imputation methods exploiting LD are based on the assumption that individuals sharing alleles at one marker will also tend to share alleles at linked SNPs. Thus, missing alleles of individuals genotyped with a LowD array can be imputed by identifying shared haplotypes within the reference population genotyped with a HighD array (Li *et al*. 2009; Marchini & Howie 2010). Pedigree‐based methods make inferences between closely related individuals to identify shared haplotypes (Antolín *et al*. 2017) and thus provide an alternative source of information when the genotyped SNPs are not sufficiently informative.

In the only previous study of imputation in dogs, Friedenberg & Meurs (2016) analysed the imputation of genotypes from a SNP array to whole‐genome sequence (WGS) data in dogs, focusing on the reference panel composition. However, the canine HighD array remains expensive and thus limits the potential sample size of genomic studies. Therefore, it is valuable to also assess the accuracy of genotype imputation from a hypothetical LowD array to the HighD array with the aim of developing a cost‐effective genotyping strategy for GWAS and genomic selection. Considering the acknowledged genetic structure and inbreeding within dog breeds (Lindblad‐Toh *et al*. 2005) and the fact that, compared to livestock species, pedigree information and genotyped ancestors are less available in dogs, it is of interest to evaluate the influences of key factors on imputation accuracy. These factors include the influence of marker density of the LowD array, the relationships between dogs in the study and the use of pedigree information.

The aim of this study was to analyse the effects of genetic and pedigree characteristics on the accuracy of genotype imputation in Labrador Retrievers using simulated scenarios based on a real dataset. Furthermore, this study provides first insights on how the application of imputed genotypes would influence GWAS and genomic prediction in dogs.

## Methods

### Dataset

The dataset of genotyped dogs for this analysis comprised 1179 Kennel Club registered (purebred) Labrador Retrievers from the UK and has previously been used for studies of complex traits (e.g. Sánchez‐Molano *et al*. 2014a,b). The pedigree structure for these dogs had the following features: the genotyped dogs were offspring of 725 sires (1.63 dogs per sire) and 1069 dams (1.10 dogs per dam), four of these sires and 22 of these dams were genotyped and included in the dataset (such that 32 dogs had at least one parent genotyped), 547 dogs were half‐siblings and 131 dogs were full‐siblings. The remaining dogs (*n *=* *501) shared no close relatives (e.g. parents or siblings) within the dataset. The genomic relationship between dogs in the validation and in the reference sets was calculated for every scenario using gemma (Zhou & Stephens 2012). For every dog in the validation set, the average and the maximum genomic relatedness with the dogs in the reference set were calculated (Table S1). It was previously demonstrated that the population of Labrador Retrievers used in this study reflects the overall genomic diversity of the UK population (Wiener *et al*. 2017).

### Genotyping and quality control

The dogs were genotyped with the Illumina Canine High Density BeadChip, which comprises 173 662 SNPs. Filtering and quality control are described by Sánchez‐Molano *et al*. (2014a). Briefly, SNPs with a call rate less than 98.4%, reproducibility (GenTrain score) less than 0.6, low or confounded signal characterised by AB R mean (mean normalized intensity of the AB cluster) less than 0.3, a minor allele frequency (MAF) less than 0.01 and deviations from Hardy‐Weinberg equilibrium were discarded. To reduce computational time, we carried out genotype imputation for different scenarios for 5826 filtered SNPs on chromosome 1 (CFA1), which is the largest autosome and has the highest gene content (122.68 Mb, 2078 genes; NCBI Annotation Release 105). To analyse the application of genotype imputation in GWAS and genomic prediction, 106 282 filtered markers for the whole genome were used.

### Genotype imputation

fimpute (version 2.2) software (Sargolzaei *et al*. 2014) was used to perform the imputation of missing genotypes. fimpute first imputes missing genotypes by using pedigree information. If no pedigree information is provided or the imputed genotypes are not inferred, population information is used to construct haplotypes by an overlapping sliding window approach. This approach takes relatedness information into account by adjusting the window sizes from long to short segments to capture distant relationships. The constructed haplotypes are then used for inferring the missing genotypes. The default settings of fimpute (shrink factor of 0.150 and overlap of 0.650 of the sliding windows) were used for this study. Imputation accuracy was calculated using the most likely genotypes estimated by fimpute for all missing genotypes.

### Scenarios for imputation

To analyse the effects of genetic and pedigree characteristics on the performance of genotype imputation, scenarios were designed based on four criteria and are summarised in Table 1. The scenarios were based on four approaches:

**Table 1:** Overview of scenarios

Name	*n*_Ref_/*n*_Val_	SNPs_masked_ (%)	Pedigree	Reference set
Ref90	1062/117	87.5	Yes	Random
Ref90NoPed	1062/117	87.5	No	Random
Ref50	590/589	87.5	Yes	Random
Ref50NoPed	590/589	87.5	No	Random
Ref10	117/1062	50–98.4^a^	Yes	Random
Ref10NoPed	117/1062	87.5	NO	Random
REL	206/454	87.5	Yes	Systematic
REL‐C	206/454	87.5	Yes	Random

*n*_Ref_, number of dogs in the reference set; *n*_Val_, number of dogs in the validation set; SNPs_masked_, proportion of SNPs in the high‐density array that were masked to generate the low‐density array; random, dogs randomly grouped into reference and validation sets; systematic, dogs in the validation set with at least one half‐sibling in the reference set.

a For Ref10, masking of SNPs was step‐wise increased by 50% to generate multiple low‐density arrays with 50%, 75%, 87.5%, 93.8%, 96.9% and 98.4% masked SNPs.

1. Size of the reference population: 10% (‘Ref10’), 50% (‘Ref50’) and 90% (‘Ref90’) of the dogs were randomly assigned to the reference population, and the remainder was assigned to the validation population.
2. Use of pedigree information in imputation: Scenario 1 was repeated without pedigree information (‘Ref10NoPed’, ‘Ref50NoPed’ and ‘Ref90NoPed’ respectively).
3. Relatedness between the reference and validation population: Only full‐sibs and paternal half‐sibs were used (*n *=* *660). One offspring of each sire was randomly selected for the reference population, resulting in 206 dogs in the reference population and 454 dogs in the validation population (scenario ‘REL’). As a control, the same number of dogs as in REL was randomly sampled from the full dataset without considering pedigree information (‘REL‐C’).
4. SNP density of the LowD array: Ref10 was repeated for varied SNP densities on the LowD array. LowD arrays were simulated masking 50%, 75%, 87.5%, 93.8%, 96.9% and 98.4% SNPs as missing, corresponding to consecutively halving the number of non‐masked SNPs.

For all scenarios, the reference population was genotyped with the HighD array comprising the 5826 SNPs on CFA1. For the first three scenarios (1, 2 and 3) the SNPs from the LowD array were masked as missing at 87.5% of the HighD array's SNPs. This was done by coding all SNPs as missing except every eighth SNP, when ordered by their position on CFA1. For these scenarios, the number of markers on the LowD array was similar to the number of markers on CFA1 on the 22K array that was previously available for dogs. The same masking procedure was applied for approach 4 but for different masking densities (e.g. coding every second SNP as missing for 50% masking density and coding all SNPs as missing except every fourth for 75% masking density). Statistics describing the distribution of marker density on the HighD and LowD arrays are provided in Table S2.

For validation of imputation, 10 replicates were performed for all scenarios, except for REL, for which the dogs were randomly reassigned to either the reference or validation population and imputation was repeated. Each dog was used in the validation population a different number of times for the particular scenarios, for example, once out of 10 replicates for Ref90, five times for Ref50 and nine times for Ref90.

Further, an analysis of variance was run for the measures of imputation accuracy to test for the effect of relatedness by comparing the REL to the REL‐C scenarios.

### Measures of imputation accuracy

The percentage of correctly imputed genotypes (‘% correct’) and the correlation between true and imputed genotypes (‘corr’) were computed to evaluate the success of imputation. The % correct was the proportion of correctly imputed genotypes out of all imputed SNPs. The corr measure was calculated using the R package sicurracy version 0.3.2 (Edwards 2017) as the Pearson correlation between the true genotypes and the imputed discrete genotypes (0, 1 or 2). True and imputed genotypes were standardised (by subtracting the mean allele frequency divided by the standard deviation) to correct for MAF, as proposed by Bouwman *et al*. (2014), with allele frequencies estimated from the true genotypes of the validation population. The % correct and corr were computed for each dog (animal‐wise accuracy) and then averaged across all dogs in a scenario.

To evaluate the effect of MAF on imputation accuracy, corr was also calculated for each SNP (SNP‐wise accuracy averaged across all dogs in a scenario). The SNPs were binned according to their MAF, as estimated in the HighD dataset, into the following MAF bins: [0, 0.025), [0.025, 0.05), [0.05, 0.075), [0.075, 0.1), [0.1, 0.2), [0.2, 0.3), [0.3, 0.4) and [0.4, 0.5), as described by Hickey *et al*. (2012). The measure % correct has been shown to overestimate imputation accuracy for SNPs with low MAF (Hickey *et al*. 2012; Calus *et al*. 2014) and therefore was not provided for this analysis.

### Application of imputed genotypes

A GWAS for the hip‐dysplasia‐related trait Norberg Angle right (NA_right), as described by Sánchez‐Molano *et al*. (2014a), and the genomic prediction of the same trait, as described by Sánchez‐Molano *et al*. (2015), were repeated using imputed genotypes for the whole genome. Therefore, 87.5% of the 106 282 markers were masked in 90% of the dogs to simulate the Ref10 scenario. The GWAS was carried out using a linear mixed model in gemma (Zhou & Stephens 2012) (for more information see Sánchez‐Molano *et al*. 2014a). To validate differences between the GWAS using real genotypes (GWAS_real_) and the GWAS using imputed genotypes (GWAS_imputed_), the correlation between the *P*‐values and the effect size of every marker was calculated. To estimate the breeding value with imputed genotypes of the same trait, the GBLUP method was applied using acta (Gray *et al*. 2012) (for further information see Sánchez‐Molano *et al*. 2015). Here, to validate differences between estimating breeding values (EBVs) with true and imputed genotypes, measures of the accuracy of genomic prediction (correlation of the predicted EBV with phenotype averaged over five validation sets, *r*; predictive abilities of EBVs, PA) were calculated for the imputed dataset and compared to that for the real dataset.

## Results

### Size of the reference population

The scenario with the largest reference population (Ref90) resulted in the highest percentage of correctly imputed genotypes (% correct = 98.6%) and highest correlation between true and imputed genotypes (corr = 0.95) when compared to scenarios with smaller reference populations (Ref50 and Ref10). Reducing the size of the reference population reduced % correct to 98.4% and 97.4% for Ref50 and Ref10 respectively (Table 2). The corr statistic was reduced similarly to 0.94 and 0.92 for Ref50 and Ref10 respectively (Table 2).

**Table 2:** Animal‐wise imputation accuracy by scenario

Scenario	Proportion of correctly imputed genotypes (% correct)^a^		Correlation between true and imputed genotypes (corr)^a^	
	Average	SD	Average	SD
Ref90^b^	98.626	1.677	0.948	0.078
REf90NoPed^b^	98.553	1.674	0.946	0.078
Ref50^b^	98.390	1.819	0.939	0.088
Ref50NoPed^b^	98.315	1.817	0.938	0.088
Ref10^b^	97.432	2.359	0.916	0.095
Ref10NoPed^b^	97.373	2.351	0.915	0.095
REL^c^	98.792	1.213	0.972	0.035
REL‐C^c^	97.668	2.265	0.926	0.086

a Statistics were calculated across all 10 replicates for the particular scenarios except for REL, for which there were no replicates.

b Dogs were randomly grouped into the reference and the validation sets, and 87.5% of genotypes were masked in the high‐density array to generate the low‐density array; Ref90, 90% of dogs in the reference set; Ref50, 50% of dogs in the reference set; Ref10, 10% of dogs in the reference set; NoPed, indicates that the imputation of the particular variant was run without pedigree information.

c Dogs in the reference set (31%) had at least one half‐sibling in the validation set (REL; 69%). The REL‐C controls had the same number of dogs as REL, but dogs were selected at random for the reference and the validation sets. In REL and REL‐C, 87.5% of genotypes were also masked in the high‐density array to generate the low‐density array.

### Pedigree information

When imputation was carried out without pedigree information, both % correct and corr were reduced by a very small amount for all scenarios (Table 2). For the scenario Ref10 for example, correct % was reduced from 97.43% to 97.37% and corr was reduced from 0.916 to 0.915.

### Relatedness between reference and imputation population

Using the reference set of full‐ and half‐sibs (REL) increased % correct by 1% and increased corr from 0.926 to 0.972 compared to the control (REL‐C) (Table 2, Table S3). The difference between imputation accuracy (% correct and corr) for REL and the 10 REL‐C replicates was significant (*P *<* *0.001). Moreover, corr (0.97) and % correct (98.8%) were higher for the REL scenario than for all other scenarios analysed in this study.

### SNP density of the LowD array

**Figure 1:**
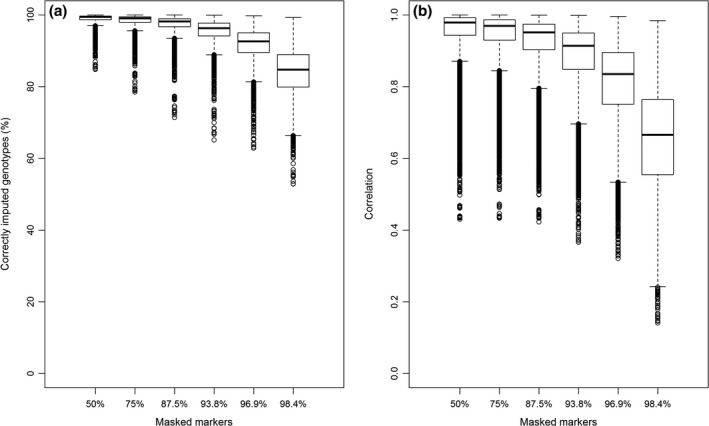
Animal‐wise imputation accuracy vs. SNP density of the low‐density array. Boxplots (maximum, 75% quartile, median, 25% quartile, minimum) show animal‐wise accuracy measurements: (a) the correctly imputed genotypes (% correct) and (b) correlation between true genotypes and imputed genotypes (corr) vs. different levels of masking of the high‐density array to generate the low‐density array (for which 10% of dogs were randomly grouped into the reference set and the remaining 90% into the validation set, scenario Ref10).

Decreasing the SNP density of the LowD array decreased both % correct and corr of the imputed genotypes (Fig. 1a,b). In the figure, boxplots of each dog's % correct (Fig. 1a) and corr (Fig. 1b) values under different SNP densities of the LowD array are depicted. The highest LowD array density (50%) yielded the highest % correct (98.9%) and corr (0.94), and this decreased to 84.1% and 0.65% respectively for the 98.4% density. The reduction of imputation accuracy was more severe when the proportion of masked SNPs was 93.5% or above.

**Figure 2:**
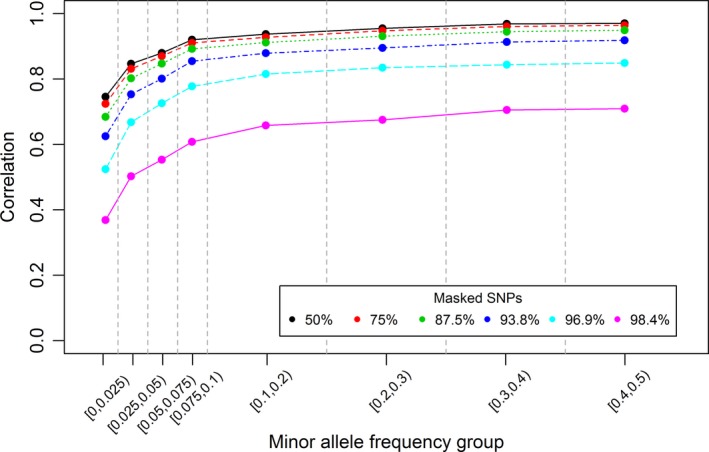
Marker‐wise correlation between true genotypes and imputed genotypes vs. the minor allele frequency of masked SNPs for different proportions of masked SNPs in the low‐density array (for which 10% of dogs were randomly grouped into the reference set and the remaining 90% into the validation set, scenario Ref10).

The corr statistic increased with increasing MAF for all LowD array densities (Fig. 2). In the figure, the average corr when calculated for SNPs in different MAF bins are depicted. The greatest increase was observed between MAF bins [0, 0.025) and [0.025, 0.05).

### Application of imputed genotypes

**Figure 3:**
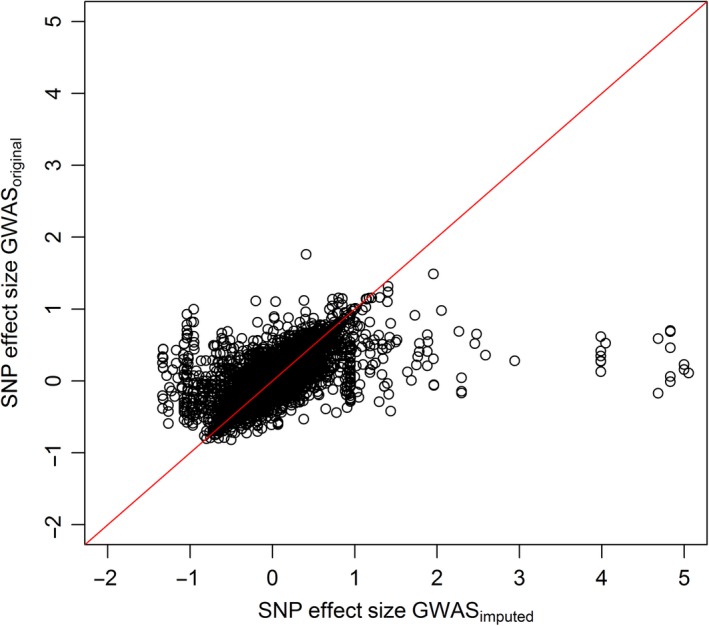
Effect sizes of SNPs for the trait Norberg Angle right calculated by a GWAS using the true genotypes (GWAS_real_) and imputed genotypes (GWAS_imputed_).

Genome‐wide imputation accuracy for the Ref10 scenario with 87.5% masked genotypes was 0.93 ± 0.04. The correlation of *P*‐values between the GWAS_real_ and GWAS_imputed_ for NA_right was 0.824. The effect sizes for the SNPs of the GWAS_real_ and GWAS_imputed_ for this trait also showed high concordances (*r *=* *0.741) with a few outliers (Fig. 3). All outlier SNPs had a very low MAF (between 0.01 and 0.015). The accuracy of the EBV for NA_right was *r *=* *0.147 and PA = 0.273 compared to *r *=* *0.145 and PA = 0.272 for the real data reported by Sánchez‐Molano *et al*. (2015).

## Discussion

In this study, we analysed different parameters with potential influence on the accuracy of genotype imputation in Labrador Retriever dogs. Results showed high imputation accuracies, even for high levels of masking on the LowD array; for example, when masking 87.5% of the SNPs on the HighD array, the percentage of correctly imputed genotypes (% correct) ranged from 97.4% (Ref10NoPed) to 98.8% (REL) and the correlation between true and imputed genotypes (corr) ranged from 0.92 (Ref10NoPed) to 0.97 (REL).

### Pedigree information and relatedness

We expected imputation with pedigree to be more accurate considering that fimpute is a program that exploits pedigree information (Sargolzaei *et al*. 2014). Accordingly, imputation without pedigree (Ref10NoPed, Ref50NoPed and Ref90NoPed) resulted in lower imputation accuracies across scenarios in contrast to their counterparts with pedigree (Ref10, Ref50 and Ref90); however, the difference was very small for all scenarios. Similar observations have been made in cattle, for which accuracies calculated for imputations without pedigree information were at least as good as for imputations with pedigree (Boison *et al*. 2015). In datasets with a large number of unrelated animals, as was the case in the current study, the information provided by using pedigree information is presumably very limited. This finding is particularly interesting for imputation in dogs: information about relatedness may be missing in non‐registered pets, but those dogs might still be useful for a genetic study because they express interesting phenotypes or are carriers of a rare disease.

In another scenario of this study (REL), relatedness between dogs in the reference and validation populations was maximised. Genotype imputation in the REL scenario performed better than in all other scenarios. This is consistent with previous studies in livestock, in which it has been shown that imputation accuracy is positively correlated with the number of genotyped ancestors (Howie *et al*. 2009; Mulder *et al*. 2012; Pausch *et al*. 2013; Bouwman *et al*. 2014; Boison *et al*. 2015; Khankhanian *et al*. 2015). Regarding relatedness, there is less family structure in dogs (even in pedigreed dogs) than is found in livestock animals; in the case of this real dataset, for which pet dogs living in normal households were recruited to analyse a specific disease, about half of the dogs had no genotyped close relatives. However, the differences in imputation accuracy between REL and all other scenarios were small, especially considering the % correct findings. These findings indicate that dogs should not be excluded from imputation due to poor kinship with the overall dataset, but it is worth selecting dogs for the reference vs. the validation set according to their relatedness to maximise imputation success.

### Design of the LowD array

Imputation accuracy decreased with decreasing SNP density on the LowD array, which is consistent with studies across livestock and crop species (Hickey *et al*. 2012; van Binsbergen *et al*. 2014; Boison *et al*. 2015). In general, the decrease of imputation accuracy with decreasing SNP density could be attributed to the greater difficulty of phasing genotypes into haplotypes in the validation population, which is less precise the fewer SNPs are available. Although a high extent of LD has been observed within various dog breeds (Lindblad‐Toh *et al*. 2005) and this population (Wiener *et al*. 2017), the difficulty of phasing with a small number of SNPs also appears to be the case for dogs.

The decrease in imputation accuracy in this study was steeper once the percentage of masked SNPs exceeded 93.8%, similar to the results from an imputation experiment in maize with the same proportions of masked SNPs (Hickey *et al*. 2012). However, it is apparently not the proportion of SNPs that need to be imputed that is important but rather the actual SNP density of the LowD array for the chromosome. For example Friedenberg & Meurs (2016) reported very high correlations in dogs between true and imputed genotypes (~0.95) for a genome‐wide percentage of missing SNPs similar to our chromosome‐wide 98.4% masking level, whereas in our study, corr reached only 0.65. What presumably led to the more accurate imputation in their study is that their LowD array had superior SNP coverage per chromosome (several thousands) in contrast to the sparse coverage of only 91 genotyped SNPs on CFA1 in the LowD array in our study after masking 98.4% of SNPs. A higher coverage of the chromosome by the LowD array improves the reconstruction of haplotypes, because in individuals that are not closely related, the shared haplotype stretches are much shorter than in related individuals (Li *et al*. 2009). Accordingly, increasing the SNP density on the LowD array (and thus chromosome coverage) yielded higher imputation accuracies in the Ref10 scenario (Fig. 1).

Results from the current study suggest that if a LowD array was designed for dogs in order to carry out genotype imputation, the number of genotyped SNPs per chromosome should not fall too low. Based on our results for CFA1, ~728 SNPs (when 87.5% genotypes are masked) could be seen as a compromise between accurate imputation and a small number of SNPs to reduce genotyping costs. This would correspond to a genome‐wide ~22K array if our results are representative of the remaining chromosomes. Further work would need to be done on optimal spacing of markers on the LowD array, as some regions (e.g. the major histocompatibility complex region) might require denser coverage than others.

Imputation accuracy also depended on the allelic diversity. Regardless of the SNP density on the LowD array in the Ref10 scenario, imputation accuracy was the lowest for SNPs with extremely low MAF (<0.025) and improved as MAF increased. It is assumed that a low MAF hinders the construction of haplotypes, as only a small number of animals are carriers of the minor allele (Heidaritabar *et al*. 2015). However, it is worth considering markers with a low MAF when designing a LowD array for dogs, because when 87.5% or fewer SNPs were masked, masked SNPs with low MAF (down to 0.05) still showed reasonable accuracies. Furthermore, it should be considered that although Labrador Retrievers were among the 28 breeds used to develop the 170K array, other breeds may be less polymorphic for these SNPs and thus imputation accuracy might be somewhat lower. If the analysis of rare variants is of interest in a genetic study, genotype imputation from HighD arrays to WGS data might be useful, as shown by Southam *et al*. (2017).

### Size of the reference population

In addition to the design of the LowD array, the number of individuals genotyped at LowD and HighD is important. Decreasing the size of the reference population from 1062 dogs (Ref90) to 117 dogs (Ref10) decreased imputation accuracy somewhat, in accordance with previous studies in livestock (Khatkar *et al*. 2012; García‐Ruiz *et al*. 2015; Heidaritabar *et al*. 2015; Moghaddar *et al*. 2015). This can be explained by a decreased number of haplotypes in the reference population that overlap with the haplotypes in the validation population. However, the scenario with the smallest reference population (Ref10) with 87.5% masked SNPs still yielded reasonable accuracies.

### Application of imputed genotypes

We showed that the GWAS_imputed_ for the hip‐dysplasia‐related trait NA_right gave results similar to GWAS_real_, considering both *P*‐values for the association and the SNP effects. Poor concordance was shown by SNPs with a very low MAF. This observation further underpins the importance of a sufficient allele frequency for correct imputation, which further limits the use of imputed genotypes from LowD arrays in the fine mapping of complex traits (reviewed by Dreger *et al*. 2016). Instead, accurate genotype imputation from HighD arrays to WGS data, as demonstrated by Friedenberg & Meurs (2016), could be the key to cost‐effective fine mapping of complex traits. However, a commercial canine LowD array may be justified to provide a cost‐effective way of identifying genomic regions using a GWAS (with subsequent fine mapping) and implementing genomic selection, given the very high concordance between EBVs for NA_right using imputed genotypes and using real genotypes (*r *=* *0.147 and PA = 0.273 vs. *r *=* *0.145 and PA = 0.272 in Sánchez‐Molano *et al*. 2015).

### Implications

The goal of implementing genotype imputation in genetic studies is to apply an appropriate trade‐off between genotyping costs and imputation accuracy. The aim of this study was to analyse a real‐case scenario for genotype imputation in dogs and determine factors that have an influence on imputation accuracy. Although we only simulated the LowD arrays, the results provide valuable information for the design of real LowD arrays and the development of an imputation strategy for canine genetic studies.

Assuming a scenario with the commonly used SNP genotyping array for the reference population (HighD array = 170K SNPs) and an ~22K array as a LowD array for the imputed population, reasonable imputation accuracies can be reached in dogs even without pedigree information and the weaker relatedness compared to livestock animals. In accordance, dogs with no relatives or unknown pedigree should be kept in the analysis because they may provide other valuable information. Nevertheless, our results and previous findings in livestock animals suggest that genotype imputation can be improved by informed assignment of dogs into the reference and validation populations.

However, although genotype imputation from LowD arrays to HighD arrays is an appropriate approach for the identification of regions of interest in GWAS and genomic selection, genotype imputation from HighD arrays to WGS data should be considered for the dissection of complex traits and the analysis of rare variants.

## Conflict of interest

The authors declare that they have no conflict of interest.

## Supporting information


**Table S1** Average genomic relationship between dogs in the reference and validation set for every scenario. For every dog in the validation set, the average and the maximum genomic relatedness (GRmean; GRmax) with the dogs in the reference set were calculated.

**Table S2** Marker spacing (in kbp) on the HighD and LowD arrays for CFA1.

**Table S3** Animal‐wise imputation accuracy (% correct and corr) for the 10 replicates of the control scenario REL‐C.
