# Supplementary material for: Accuracy of genotype imputation in Labrador Retrievers
Source: Anim Genet. 2018 Jul 5;49(4):303–11. doi: 10.1111/age.12677 (PMC6055857; doi:10.1111/age.12677)
Supplement: Supplementary file 1 — Table S1 Average genomic relationship between dogs in the reference and validation set for every scenario. For every dog in the validation set, the average and the maximum genomic relatedness (GRmean; GRmax) with the dogs in the reference set were calculated. [file AGE-49-303-s001.pdf]

Table S1 Average genomic relationship between dogs in the reference and validation set for every scenario. For every dog in the validation set, the average and the maximum genomic relatedness (GRmean; GRmax) with the dogs in the reference set were calculated

Scenario	GRmean	GRmax
Ref10	0.04	0.13
Ref50	0.04	0.17
Ref90	0.04	0.18
REL	0.04	0.18
REL-C	0.04	0.17
