# Supplementary material for: Accuracy of genotype imputation in Labrador Retrievers
Source: Anim Genet. 2018 Jul 5;49(4):303–11. doi: 10.1111/age.12677 (PMC6055857; doi:10.1111/age.12677)
Supplement: Supplementary file 2 — Table S2 Marker spacing (in kbp) on the HighD and LowD arrays for CFA1. [file AGE-49-303-s002.pdf]

Table S2 Marker spacing (in kbp) on the HighD and LowD arrays for CFA1

Array	Masked markers (%)	n	Average	SD	Min	Max	Range
HighD	0	5826	21	23	0.02	486	486
LowD	50	2913	42	35	0.84	531	530
LowD	75	1457	84	53	8.32	550	542
LowD	87.5	728	168	85	57.96	730	672
LowD	93.8	365	336	137	152.22	909	757
LowD	96.9	183	672	238	347.00	1716	1369
LowD	98.4	92	1343	378	715.76	2969	2253
