# Supplementary material for: Accuracy of genotype imputation in Labrador Retrievers
Source: Anim Genet. 2018 Jul 5;49(4):303–11. doi: 10.1111/age.12677 (PMC6055857; doi:10.1111/age.12677)
Supplement: Supplementary file 3 — Table S3 Animal‐wise imputation accuracy (% correct and corr) for the 10 replicates of the control scenario REL‐C. [file AGE-49-303-s003.pdf]

Table S3 Animal-wise imputation accuracy (% correct and corr) for the 10 replicates of the control scenario REL-C

Control	Proportion of correctly imputed genotypes (% correct)	Correlation between true and imputed genotypes (corr)
REL-C1	97.699	0.926
REL-C2	97.575	0.924
REL-C3	97.585	0.923
REL-C4	97.747	0.928
REL-C5	97.609	0.924
REL-C6	97.732	0.926
REL-C7	97.717	0.928
REL-C8	97.826	0.933
REL-C9	97.603	0.924
REL-C10	97.587	0.925
